# In-line coupling of capillary-channeled polymer fiber columns with optical absorbance and multi-angle light scattering detection for the isolation and characterization of exosomes

**DOI:** 10.1007/s00216-024-05283-z

**Published:** 2024-04-09

**Authors:** Sarah K. Wysor, R. Kenneth Marcus

**Affiliations:** Department of Chemistry, Biosystems Research Complex, Clemson University, Clemson, SC 29634-0973 USA

**Keywords:** Extracellular vesicles (EVs), Exosomes, High-performance liquid chromatography (HPLC), Capillary-channeled polymer (C-CP) fibers, Multi-angle light scattering (MALS)

## Abstract

Extracellular vesicles (EVs) have garnered much interest due to their fundamental role in intracellular communication and their potential utility in clinical diagnostics and as biotherapeutic vectors. Of particular relevance is the subset of EVs referred to as exosomes, ranging in size from 30 to 150 nm, which contain incredible amounts of information about their cell of origin, which can be used to track the progress of disease. As a complementary action, exosomes can be engineered with therapeutic cargo to selectively target diseases. At present, the lack of highly efficient methods of isolation/purification of exosomes from diverse biofluids, plants, and cell cultures is a major bottleneck in the fundamental biochemistry, clinical analysis, and therapeutic applications. Equally impactful, the lack of effective in-line means of detection/characterization of isolate populations, including concentration and sizing, is limiting in the applications. The method presented here couples hydrophobic interaction chromatography (HIC) performed on polyester capillary-channeled polymer (C-CP) fiber columns followed by in-line optical absorbance and multi-angle light scattering (MALS) detection for the isolation and characterization of EVs, in this case present in the supernatant of Chinese hamster ovary (CHO) cell cultures. Excellent correlation was observed between the determined particle concentrations for the two detection methods. C-CP fiber columns provide a low-cost platform (< $5 per column) for the isolation of exosomes in a 15-min workflow, with complementary absorbance and MALS detection providing very high-quality particle concentration and sizing information.

## Introduction

Extracellular vesicles (EVs) have become increasingly relevant as the fundamental understanding of their roles in diverse cellular processes has evolved [1, 2]. EVs have different subsets, typically defined based on their size, including apoptotic bodies, microvesicles, exosomes, and exomeres [3, 4]. Exosomes, or small EVs (sEV), range in size from 30 to 150 nm in diameter, and were originally believed to be used for cellular dumping. It has been discovered that exosomes encapsulate information, such as proteins and diverse genetic material, while their vesicular surfaces are decorated with membrane proteins from their host cell of origin. Moreover, the source of EVs influences their composition and functions after secretion [3, 5]. Due to their ability to encase information, exosomes have been proven to participate in cell-cell communication. Their surface markers play a role in the direct targeting of specific cells [4], and in this way can also serve as a mode of diagnosis due to their presence in biofluids such as urine, blood, and saliva [6]. Ultimately, the processes which drive their role in intracellular communication and the potential to serve as payload carriers open avenues for exosomes as potential therapeutic vectors [7, 8].

Due to the plethora of natural and cell culture exosome sources, along with their potential to hold diverse and relatively large cargo payloads, they exhibit great potential as therapeutic vectors [7–10]. In comparison to more evolved vector systems such as adeno-associated viruses (AAV) [11] and lipid nanoparticles [12, 13], exosomes show particular promise due to their natural origin achieving better immune responses, where AAVs can host immune responses [14] and the synthetic nature of lipid nanoparticles can promote immune responses, disrupt cell development, or are unstable in vivo [15, 16].

With the potential for implementation as vectors, the ability to effect high-efficiency isolation, purification, and characterization of EVs is essential, with the intent to move towards large-scale isolations presenting further challenges in terms of practicality. However, their isolation is the current bottleneck in all sectors and scales of exosome study and application, as current methods are costly and time consuming, result in low purities, and are of limited capacity [17–21]. Common isolation techniques of EVs include ultracentrifugation (UC) [22, 23], ultrafiltration (UF) [24, 25], immunoaffinity capture [26, 27], and polymer precipitations [3, 19, 28]. UC is a density-based isolation that is easily implemented; however, it often results in low exosome purities, requires large sample volumes, and has high capital costs [20, 29–31]. UF relies upon differences in hydrodynamic volumes to isolate exosomes, with high purities, but the filters/pores are prone to clogging, resulting in the loss of product [18, 32, 33]. Related in terms of separation based on hydrodynamic volumes, size exclusion chromatography (SEC) (though not operating in an HPLC mode) provides fairly rapid processing, though here differentiation of species in the size domain of sEVs can be ambiguous [34, 35]. Immunoaffinity capture approaches target the specific biomarkers on the outer bilayer of EVs, yielding very high purities, but very low overall yields of a singular population of exosomes and presenting very high consumable costs [17, 19, 36]. With polymer precipitation, a polymer and salt solution is used to reduce the solubility and precipitate the EVs from the solution [18]. This method results in a high yield, but low purity due to other matrix components (i.e., extracellular proteins and protein aggregates) co-precipitating with the exosomes [37]. While each of these isolation approaches has its positive and negative attributes, a method that yields high-purity EVs from diverse matrices is also readily automated, and provides integrated means of detection/characterization that would be impactful in the arena.

In an effort to improve the overall processing exosomes, Marcus et al. have successfully isolated/purified exosomes using hydrophobic interaction chromatography (HIC) performed on polyester (PET) capillary-channeled polymer (C-CP) fiber chromatography columns [38–41] and spin-down tips [42–45]. EVs from various sources ranging from biofluids (urine, plasma/serum, breast milk, etc.) to plants (blueberries, tomatoes, etc.) and cell culture media have been demonstrated [43–48]. The hydrophobicity-based separations are nondenaturing as they rely on high salt concentrations for binding conditions and low salt concentrations for elution. Previous efforts by this lab have also shown the addition of organic modifiers to elute exosomes without affecting the vesicular structure of the isolated exosomes [38, 42, 49]. Direct comparisons between the C-CP fiber separations with most of the previously cited approaches have revealed higher overall yields, greater purities, lower overhead costs, and greater throughput for the fiber-based separations [42].

The characterization of isolated exosomes is also necessary regardless of their future use, whether fundamental biochemical data, clinical diagnostics, or vector development. Characterization can entail many different biological, chemical, or physical attributes, including concentration, particle size distributions, isolate purity, surface protein identification, or genetic content. One would likely consider concentration and particle size distribution to be a key set of first-level characteristics. Current characterization techniques include absorbance, fluorescence, nanoparticle tracking analysis (NTA), and flow cytometry [3, 19]. The current quantification methods of EVs often depend on the method of isolation, including any specific solvents/media. Beyond this, the vast majority of the methods occur off-line from the isolation process, encompassing a completely different set of unit operations and instrumentation. Taken a step further, they typically require some additional sample preparation/modification. While exquisite levels of selectivity can be achieved using approaches based on immunoaffinity labeling, these methods are usually so specific and expensive as not to be practical unless absolutely warranted [50]. The most general, and indeed most widely applied, means of exosome counting/sizing is NTA [51–54]. The off-line method relies on Brownian motion and light scattering to determine the sizing and concentration, based on the statistics of random motion and the number of events. While the most common approach, the method is very susceptible to contamination from sample and ambient particles and various instrument instabilities. While the current standard, the method’s poor precision and accuracy, high capital costs, and off-line nature make it unacceptable for high-throughput applications. More recently, multi-angle light scattering (MALS) has been used for the sizing and counting of EVs [55–57]. Of particular importance here is the fact that MALS is naturally adapted to flowing (solution) systems, though there are certain compatibility issues to be addressed. As for all of the common characterization techniques, low purity samples and/or aggregation can hinder or falsely represent the results [58]. As a final means of particle quantification, Marcus and co-workers have demonstrated that simple optical absorbance detection (technically scattering) can be employed to generate standard Beer’s law plots that are well behaved over common exosome concentrations. In this case, measurements in the 200–220 nm region of the spectrum are clearly impacted by the presence of residual species such as proteins. Following separations on the C-CP fiber phases, quantitative values are in good agreement with prepared standards and NTA determinations. In comparison to other means of quantification, absorbance detection offers ready in-line implementation and the ability to use calibration functions (i.e., Beer’s Law) prepared from standards. Ultimately, it must be emphatically stated that the precision/accuracy of any form of exosome quantification (and sizing as well) is only as good as the purity of the particles that are isolated. Here the optical absorbance and MALS scattering will be evaluated and compared for the quantification of EV eluate.

Here we present the isolation and quantification of EVs using capillary-channeled polymer (C-CP) fiber stationary phases for the isolation of EVs coupled with in-line absorbance and MALS detection, providing both quantitative and sizing characterization of CHO cell–derived exosomes. While previous studies have demonstrated the utility of the C-CP fiber platform for EV isolation from diverse matrices, the focus here is on the detection methods and not the analytical matrix, which should have no bearing on the respective detector performance. The interfacing takes place without any modification of the previously developed HIC elution strategy, with a 100-mL aliquot of the CHO cell supernatant processed in less than 15 min. Implementation of MALS detection is undertaken using silica nanoparticles to calibrate both the particle count and sizing. Tandem use of absorbance and MALS detection provides excellent correlation in terms of the quantitative responses with increases in sample loading, effectively validating the two methods as well as the overall purity of the C-CP fiber column isolation process. Comparison across five separate C-CP columns is presented to confirm the reproducibility of the purification/detection protocol. It is believed that the ability to couple high-throughput chromatographic exosome isolation and in-line absorbance/MALS detection provides a powerful approach to exosome characterization.

## Materials and methods

### Chemicals and reagents

Ammonium sulfate (Thermo Scientific, MA, USA), Gibco phosphate-buffered saline (PBS) solution 10× pH 7.4 (Thermo Scientific, MA, USA), glycerol (VWR, PA, USA), acetonitrile (ACN) (VWR, PA, USA), and deionized water (DI-H_2_O) from an Elga PURELAB flex water purification system (18.2 MΩ-cm, Veolia Water Technologies, High Wycombe, UK) were used for mobile phase preparations. A silica-bead standard (QS2503 (230-260 nm), NanoFCM Nottingham, UK), was used to confirm the MALS particle count and sizing measurements. Chinese hamster ovary (CHO) cell supernatant samples obtained from the Harcum Lab (Department of Bioengineering, Clemson University) were used for all separations. Following collection from the bioreactor, the supernatant was centrifuged at 1000 × g and stored at −20 °C, before use. On the day of the separations, the supernatant was filtered with a 0.22-μm polyethersulfone (PES) filter. As a benchmark, the concentration of the IgG product in the supernatants was independently determined to be 0.71 mg mL^−1^.

### Column preparations

The preparation of the polyester (PET) C-CP fiber columns has been previously described in detail [59]. Simply, the fibers removed from the primary spool are pulled through 0.76-mm inner diameter polyether ether ketone (PEEK) tubing and trimmed to 30 cm. In this case, the total number of fibers was ~450, determined to obtain an interstitial fraction of ~0.6 [59]. Following packing, the column/fibers are washed with DI, ACN, and DI to remove any electrostatic (spin) coatings or contaminants introduced during packing. Five separate C-CP columns were prepared for this study, with fiber masses within the columns averaging 0.109 g, with a 2.4% RSD.

### Instrumentation

A Dionex Ultimate 3000 (Thermo Fisher Scientific, Sunnyvale, CA, USA) quaternary pump equipped with a diode array detector was used for all chromatographic measurements. The instruments were controlled with Chromeleon 7. A 0.22-µm polyvinylidene fluoride (PVDF) membrane inline column filter was placed between the pump and injector to remove any mobile phase contaminants. A MALS detector (Dawn, Wyatt, Nottingham, UK) was used for particle concentration and sizing of exosomes utilizing 18 separate detectors. For the MALS measurements, the parameters were set as follows: refractive index (h) for 50% glycerol in 1X PBS = 1.4096, dh/dc = 0.1850 (commonly used for proteins), and the sphere real RI h = 1.61 (Wyatt database). Particle sizing was determined using the Zimm approximation, as used in other exosome applications [55].

### Methods

The solvents for the separation included a mobile phase (MP) A of 2 M ammonium sulfate in 1X PBS and MP B of 50% glycerol in 1X PBS. A step gradient method was performed with the sample injection in 100% MP A (0–4 min) for exosome and protein binding, while sugars, salts, and small polar molecules were unretained. A step to a 50:50 mixture of MP A:MP B was employed to elute any proteinaceous species and was held for 4–10 min. Finally, a step to 100% MP B was performed (10–15 min) to elute the target EVs. Chromatographic measurements were taken in triplicate with an injection volume of 100 µL and a mobile phase flow rate of 0.5 mL min^−1^. Eluting species passed first through the system absorbance detector (216 nm) and then to the MALS detector volume. Absorbance-based assessments of exosome particle number densities were determined through calibration functions as described previously [40], while number densities via MALS were determined through serial dilution of the silica-bead standard.

## Results and discussion

### Repeatability of exosome determinations based on standard absorbance responses

**Fig. 1:**
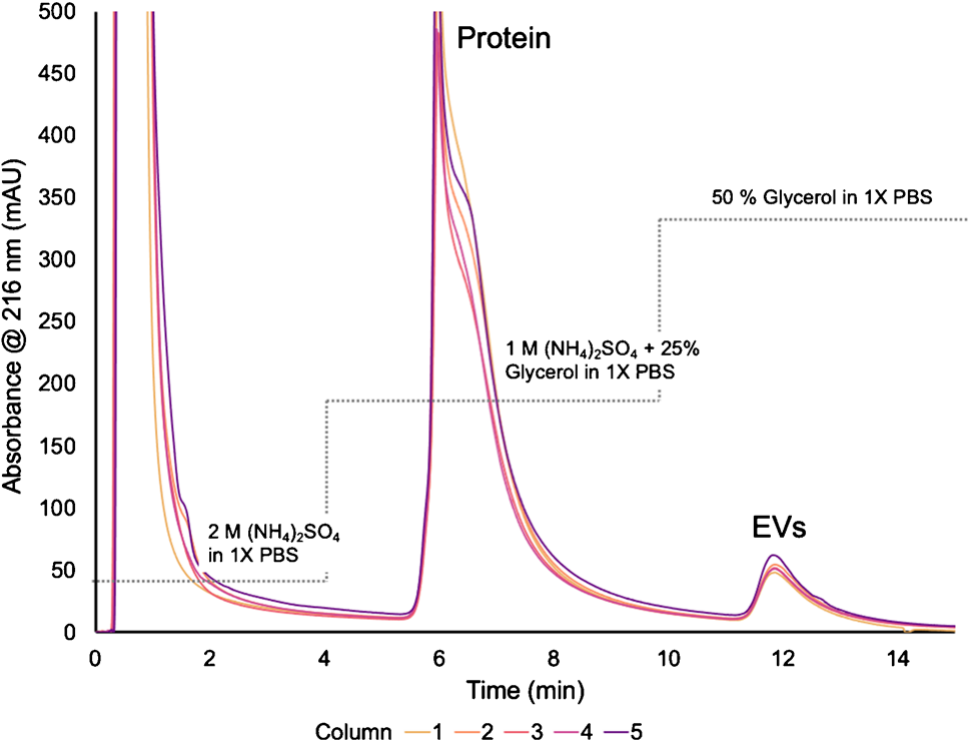
Overlay of averaged HIC separations across 5 PET C-CP columns. Absorbance is measured at 216 nm, where 2 M ammonium sulfate (A.S.) is introduced from 0 to 4 min and unretained species flow through. From 4 to 10 min, a step is taken to 1 M A.S. and 25% glycerol in 1X PBS where proteins are eluted. Finally, 50% glycerol in 1X PBS is introduced from 10 to 15 min, and EVs are eluted

While CHO cell lines are commonly known for their production of antibodies, the cells also secrete exosomes as a natural by-product of the cell metabolism [10, 46, 60, 61]. The workflow presented here aims to purify the exosomes from CHO supernatant and determine the concentration and sizing through in-line absorbance and MALS. The separations of the supernatant using PET C-CP fibers were performed in triplicate on five separate columns, with the average chromatogram for each column shown in Fig. 1. The large protein elution band is expected as CHO cell lines are grown for the production of immunoglobulin G (IgG), with a plethora of host cell proteins (HCPs) excreted as well. Slight variations in the total protein recoveries between each of the columns are observed, though the overall reproducibility across the columns was better than 5% RSD. That said, previous 2DLC experiments have shown coupling two C-CP columns (protein A and PET) for the successive isolation of therapeutics (IgG and exosomes) from CHO supernatant have shown a higher level of precision in absorbance [46].

**Fig. 2:**
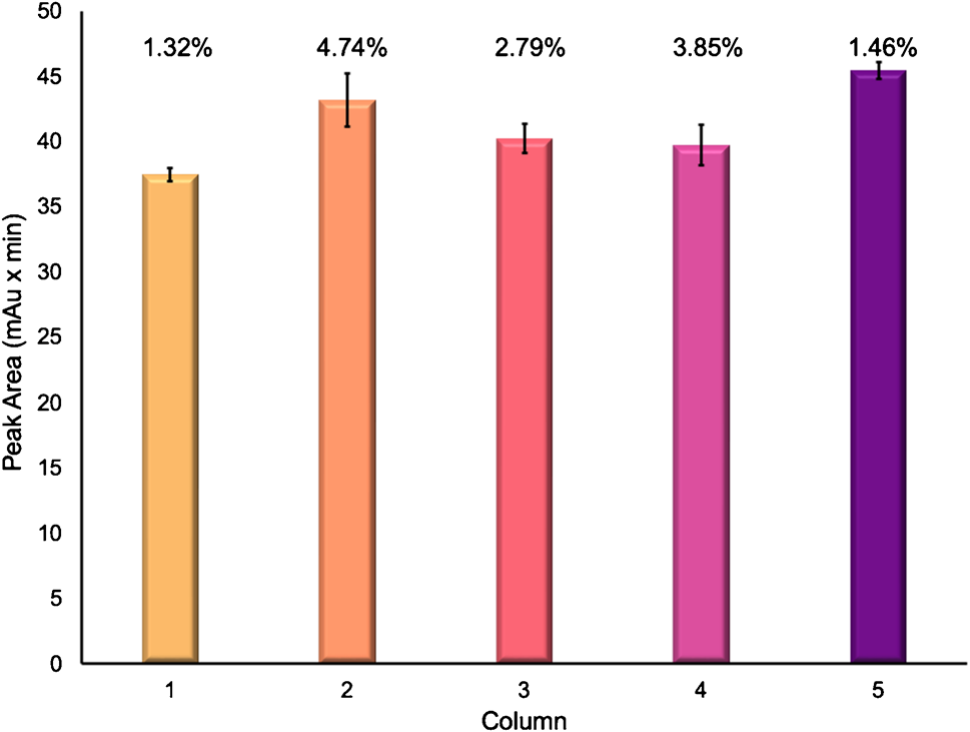
The average absorbance peak area of triplicate runs of the exosome elution fraction plotted across 5 PET C-CP columns. The text above each bar represents the %RSD

The relative process exosome recoveries, based on the integrated absorbance peak areas following the final gradient step, are presented in Fig. 2 across the five analytical columns tested. The average peak areas for the exosome fraction for each column are as follows: 37.5, 43.2, 40.2, 39.7, and 45.4 mAu × min for columns 1–5, respectively. Variations between columns are expected as the packing density between columns can vary slightly due to variations in the extruded fiber cross sections. The standard deviation of each peak area is plotted along with the percent relative standard deviation (% RSD) of triplicate runs. For each individual column, the variability was < 5% RSD, exhibiting excellent reproducibility for each column. The repeatability of the absorbance areas across all five columns (a total of 15 separations) is 7.6% RSD, while slightly higher than the intra-column variability, with excellent agreement across all five columns observed. In fact, if the absorbance response is normalized to the mass of fiber in the respective columns, the overall variability is improved to 4.9% RSD.

### MALS-determined exosome particle concentrations

**Fig. 3:**
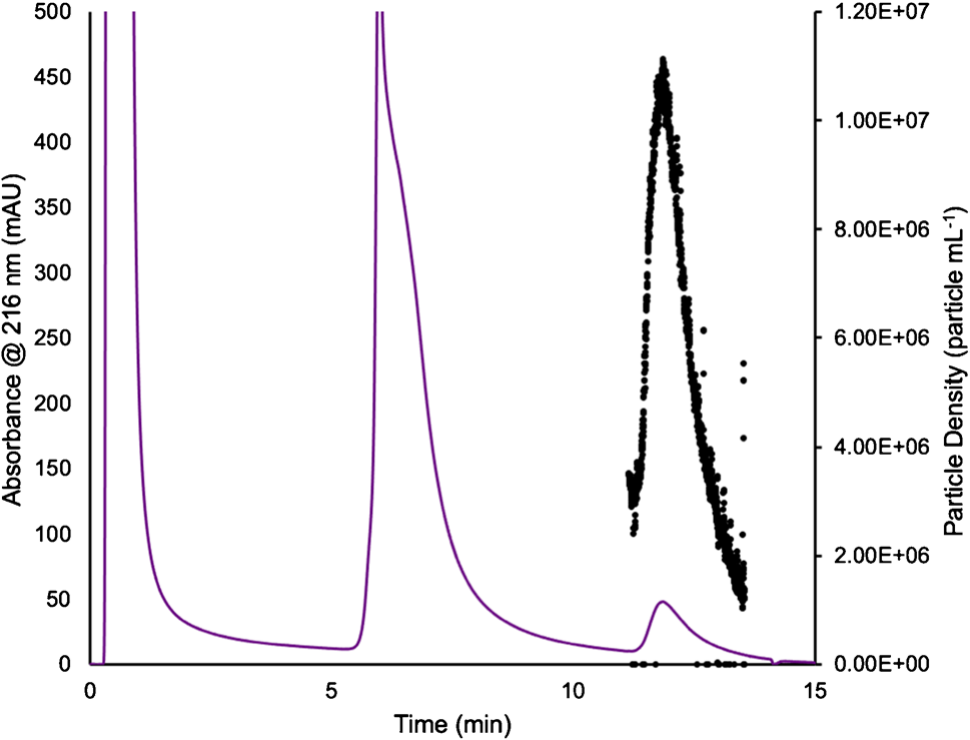
The particle density of exosomes is plotted across the elution peak. The absorbance trace (purple) at 216 nm is plotted on the left axis, while the MALS-determined particle density response (black) is plotted on the right axis

**Fig. 4:**
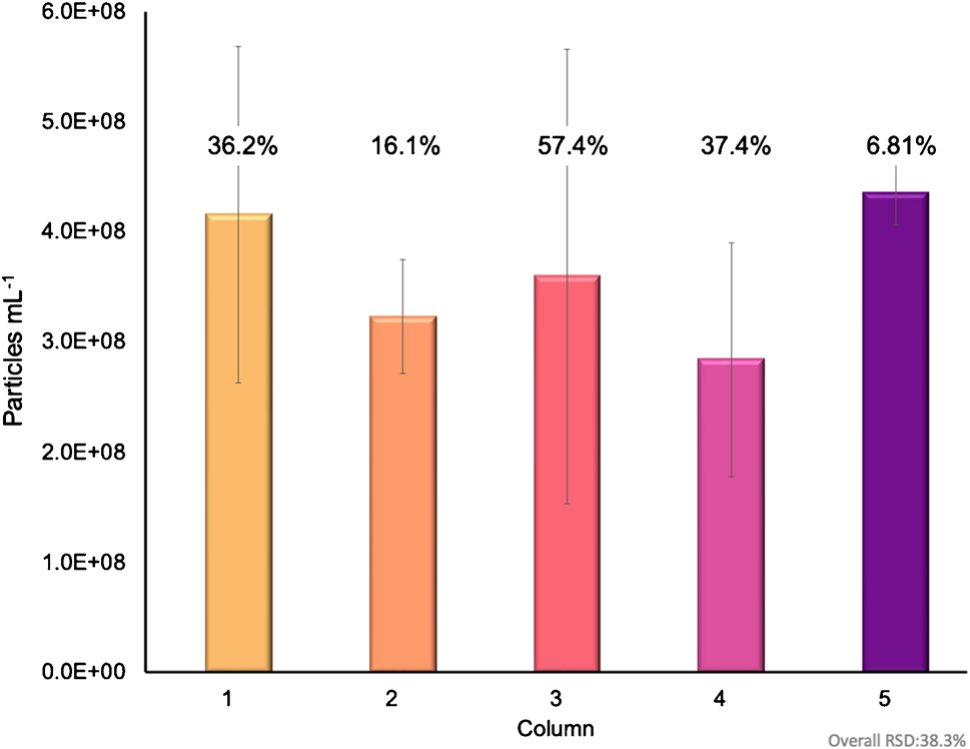
Average particle concentration of the exosome eluate across all 5 C-CP columns determined via MALS

A primary challenge in exosome characterization is accurate particle quantification. The primary challenges lie in the fact that commercial EV standards are unreliable themselves, as protein contaminants are still present within the standard, compounding the inherent variability of the actual methods of determination. As discussed previously, MALS presents an excellent alternative in terms of the quantification of particles. Following the 100-µL injections, HIC isolations, and passage through the absorbance detector, in-line MALS was used to determine the particle concentration of the EV eluates. To establish the particle concentrations, a silica standard of concentration (1–3 × 10^10^ particles mL^−1^) was used. Through the on-line MALS determination, the particle concentration was determined to be 1.70 × 10^10^ particles mL^−1^ with a 4.1% RSD for the silica particle standard, confirming the suitable operation of the detection system. An example of the MALS-determined particle densities over an exosome elution band is shown in Fig. 3, with the absorbance at 216 nm plotted on the left axis (purple) and the particle density (particles mL^−1^) plotted on the right axis (black). Across the evolving exosome elution peak, the instantaneous particle densities range from 2.0 × 10^6^ to 1.1 × 10^7^ particles mL^−1^. Of note, MALS determinations across the protein elution band were below the measurement limit of detection. Similar traces were observed across the suite of injections/columns. The area under the curve of the particle density, along with the dilution correction, was then used to determine the recovered particle concentration of exosomes, as presented in Fig. 4 [53, 62]. For the individual columns, the average, integrated exosome particle concentrations were determined to be 4.2, 3.2, 3.6, 2.8, and 4.6 (× 10^8^) particles mL^−1^ for columns 1–5, respectively, with an average value across the 15 injections of 3.7 × 10^8^ mL^−1^. Indeed, this value is in excellent agreement with the absorbance-based concentration of 3.6 × 10^8^ mL^−1^. As would be expected, the variability of the values for each column is broader than the absorbance measurements of Fig. 2, as the scattering process and the underlying assumptions in terms of the basic calculations are dependent on far more experimental variables that change in the course of the solute elution, such as the viscosity, refractive index, and particle density in the probed microvolume. That said, the precision across the whole here (38% RSD) is better than typical of NTA measurements [53, 58, 62]. As a final point, based on the integrated particle concentrations, the particles captured per mass of the fiber column resulted in ~3 × 10^9^ particles mL^−1^ g^−1^ of stationary phase, presenting an excellent opportunity for a cost-effective scale-up isolation of EVs.

### MALS-determined exosome particle diameters

**Fig. 5:**
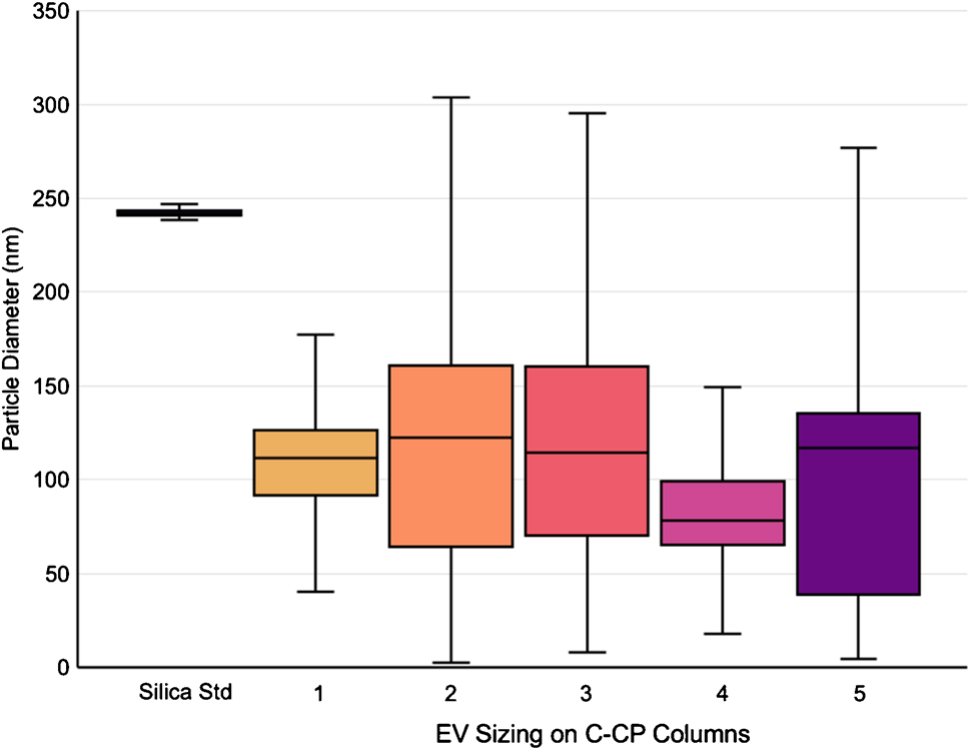
Particle sizing of silica standards and the exosome eluates determined via MALS. Triplicate runs are represented by each box and whisker for each column. The box represents the inner quartile, or 50% of the population, while the bars represent the remaining 25% on each side

Silica standards were used to verify the sizing and concentration determination reported from the MALS. Based on the use of standards of known diameters (230–260 nm), the diameter of silica determined via inline MALS was between 236 and 248 nm, consistent with the expected diameter. Along with the corresponding MALS results for the silica particles, the pooled scattering data for the CHO cell–derived EVs (three injections) for each column are presented in the box plots in Fig. 5, representing the distribution of EV diameters present in the eluate fractions. In short, the box and whisker plots entail four quartiles, each representing 25% to represent the range of determined exosome diameters. The solid box represents 50% of the data points in the inner quartiles, with the midline representing the median of the population. Of course, very different from the case of the synthetic silica beads, there are broader distributions of determined values for the naturally heterogenous EV populations [10, 53, 62]. For column 1, particle diameters were determined to range from 40 to 177 nm, with 75% falling between 40 and 126 nm. For column 2, a much wider range was observed; more importantly, the inner quartile of the population falls between 64 and 161 nm. A large overall spread was determined from column 3, again with the 50% box falling between 70 and 160 nm. The diameters of exosome eluates from column 4 were between 18 and 149 nm, with 50% falling between 65 and 99 nm. Finally, for column 5, the determined diameters were between 5 and 277 nm, with 50% between 38 and 277 nm. In each case, some of the determined sizes fall outside the range of exosomes, such as exomeres (< 50 nm) [63] and > 300 nm in diameter, which could be either aggregates or small microvesicles [64]. The differences across the columns are likely a reflection of the slight differences in the individual packing characteristics, more so than different EV populations. Overall, the most relevant values of median and 50^th^ percentile sizes were in very good agreement across all five columns, with the majority found in the expected exosome size range.

### Correlation between absorbance and light scattering responsivity

**Fig. 6:**
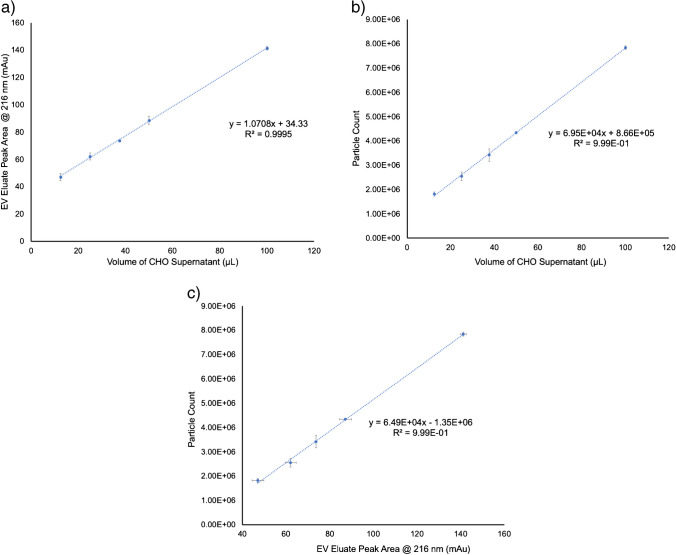
Analytical response curves as a function of CHO cell supernatant loading volume for **a** absorbance and **b** MALS detection. **c** Correlation between absorbance and MALS response curves

A primary goal of the described studies was the co-validation of the quantitative aspects of the in-house developed optical absorbance [39, 40] and MALS scattering approaches. As noted above, the absorbance and MALS-based concentrations for the CHO cell supernatant were in agreement to within 4%, absolute. In order to retain consistency, the analytical responses of the two methods were evaluated based on increasing injection volumes of the CHO cell supernatant. The primary response curves by absorbance and MALS are presented in Fig. 6a and b, respectively, for volumes ranging from 12.5 to 100 μL, a factor of 10 increase in loading. The absorbance response yielded a linear regression of *y* = 1.0708*x* + 34.33 with an *R*^2^ = 0.9995, showing outstanding linearity and high precision for the triplicate measurements at each loading volume. The significance *F* value was 5.1 × 10^6^, and the *P* value was 2.5 × 10^5^, confirming the statistical significance of the absorbance data. Based on the linear regression statistics and the variability of PBS-blank injections, the limit of detection (LOD = 3s_b_/m) was determined to be 2.6 × 10^4^ particles mL^−1^. Likewise, the MALS measurements yielded excellent linearity with a response function of *y* = 6.95 × 10^4^*x* + 8.66 × 10^5^ and *R*^2^ = 0.9994. The significance *F* value was 6.9 × 10^6^ and *P* value 5.6 × 10^4^, again confirming the validity of the approach. The LOD based on the MALS linear regression was 6.7 × 10^4^ particles mL^−1^. Here again, the standard deviations of the triplicate measurements were outstanding, as depicted by the error bars. The respective LOD values are considered to be very good in terms of potential applicability of either detection modality across diverse EV separation strategies. Ultimately, the correlation between the two responses across the 10× loading range was evaluated, as shown in Fig. 6c. A strong correlation between the absorbance and light scattering detection was observed with an *R*^2^ = 0.9985 and a linear regression of *y* = 6.49 × 10^4^*x*–1 × 10^6^. This strong correlation between the two methods provides further support of the general utility of the absorbance method as an effective means of the determination of EV concentrations.

## Conclusions

The method presented here fills a very important need in the field of exosome analytics; integrated methods of purification and characterization. Hydrophobic interaction chromatography on polyester C-CP fiber columns is coupled with standard absorbance and MALS detection. In total, a cost-effective 15-min workflow for the successful isolation, quantification, and sizing determination of EVs from CHO cell supernatant is demonstrated. The consistency of the C-CP fiber stationary phases is observed across five separate columns, along with triplicate runs in terms of absorbance recovery, particle concentrations, and particle sizing. As anticipated, the MALS sizing, while accurate in terms of the average values, does show more dispersion than the quantification responses. Importantly, the strong correlation between light scattering detection and absorbance serves to further demonstrate that simple absorbance is effective for the determination of particle count of EVs. Future works will look towards elucidating (and mitigating) the precise causes for the greater dispersion in results for MALS vs. absorbance particle density determinations, as the ability to accurately profile sizes is essential to better understanding the biochemistry of EVs. Likewise, further investigations into the respective breadth and extents of the quantitative figures of merit for both detection modalities must be determined. The combined detection methodology will be expanded across different matrices including urine and plasma. Additionally, efforts will look to scale up the C-CP columns for larger volume, preparative processing as recoveries of ~3 × 10^9^ particle mL^−1^ g^−1^ of EVs per mass of fiber column hold great promise.

## References

[1] Raposo G, Stoorvogel W (2013). Extracellular vesicles: exosomes, microvesicles, and friends. J Cell Biol..

[2] van Niel G, D’Angelo G, Raposo G (2018). Shedding light on the cell biology of extracellular vesicles. Nature Rev Mol Cell Biol..

[3] Doyle LM, Wang MZ (2019). Overview of extracellular vesicles, their origin, composition, purpose, and methods for exosome isolation and analysis. Cells..

[4] Kourembanas S (2015). Exosomes: vehicles of intercellular signaling, biomarkers, and vectors of cell therapy. Annu Rev Physiol..

[5] Anand S, Samuel M, Kumar S, Mathivanan S (2019). Ticket to a bubble ride: cargo sorting into exosomes and extracellular vesicles. Biochim Biophys Acta, Proteins Proteomics..

[6] Farzanehpour M, Miri A, Alvanegh AG, Gouvarchinghaleh HE (2023). Viral vectors, exosomes, and vexosomes: potential armamentarium for delivering CRISPR/Cas to cancer cells. Biochem Pharmacol..

[7] Sancho-Albero M, Medel-Martínez A, Martín-Duque P (2020). Use of exosomes as vectors to carry advanced therapies. Rsc Advances..

[8] Elsharkasy OM, Nordin JZ, Hagey DW, de Jong OG, Schiffelers RM, Andaloussi SEL, Vader P (2020). Extracellular vesicles as drug delivery systems: why and how?. Adv Drug Deliv Rev..

[9] Batrakova EV, Kim MS (2015). Using exosomes, naturally-equipped nanocarriers, for drug delivery. J Control Release..

[10] Zhang Y, Bi JY, Huang JY, Tang YN, Du SY, Li PY (2020). Exosome: a review of its classification, isolation techniques, storage, diagnostic and targeted therapy applications. Int J Nanomed..

[11] Bulcha JT, Wang Y, Ma H, Tai PWL, Gao G (2021). Viral vector platforms within the gene therapy landscape. Curr Signal Transduct Ther..

[12] Scioli Montoto S, Muraca G, Ruiz ME. Solid lipid nanoparticles for drug delivery: pharmacological and biopharmaceutical aspects. Front Mol Biosci. 2020;7:587997. 10.3389/fmolb.2020.587997 PMC7662460 33195435

[13] Müller RH, Mäder K, Gohla S (2000). Solid lipid nanoparticles (SLN) for controlled drug delivery – a review of the state of the art. Eur J Pharm Biopharm..

[14] Shirley JL, de Jong YP, Terhorst C, Herzog RW (2020). Immune responses to viral gene therapy vectors. Mol Ther Nucleic Acids..

[15] Mehta M, Bui TA, Yang X, Aksoy Y, Goldys EM, Deng W (2023). Lipid-based nanoparticles for drug/gene delivery: an overview of the production techniques and difficulties encountered in their industrial development. ACS Mater Au..

[16] Huang L, Wan J, Wu Y, Tian Y, Yao Y, Yao S (2021). Challenges in adeno-associated virus-based treatment of central nervous system diseases through systemic injection. Life Sci..

[17] Théry C, Amigorena S, Raposo G, Clayton A. Isolation and characterization of exosomes from cell culture supernatants and biological fluids. Curr Protoc Cell Biol. 2006;30(1):3–22. 10.1002/0471143030.cb0322s30 18228490

[18] Zeringer E, Barta T, Li M, Vlassov AV (2015). Strategies for isolation of exosomes. Cold Spring Harb Protoc..

[19] Zhang M, Jin K, Gao L, Zhang Z, Li F, Zhou F, Zhang L (2018). Methods and technologies for exosome isolation and characterization. Small Methods..

[20] Momen-Heravi F, Balaj L, Alian S, Mantel P-Y, Halleck AE, Trachtenberg AJ (2013). Current methods for the isolation of extracellular vesicles. Biol Chem..

[21] Liangsupree T, Multia E, Riekkola ML (2021). Modern isolation and separation techniques for extracellular vesicles. J Chromatogr A..

[22] Baranyai T, Herczeg K, Onódi Z, Voszka I, Módos K, Marton N (2015). Isolation of exosomes from blood plasma: qualitative and quantitative comparison of ultracentrifugation and size exclusion chromatography methods. PLoS One..

[23] An M, Wu J, Zhu J, Lubman DM (2018). Comparison of an optimized ultracentrifugation method versus size-exclusion chromatography for isolation of exosomes from human serum. J Proteome Res..

[24] Cheruvanky A, Zhou H, Pisitkun T, Kopp JB, Knepper MA, Yuen PST, Star RA (2007). Rapid isolation of urinary exosomal biomarkers using a nanomembrane ultrafiltration concentrator. Am J Physiol Renal Physiol..

[25] Xu R, Greening DW, Rai A, Ji H, Simpson RJ (2015). Highly-purified exosomes and shed microvesicles isolated from the human colon cancer cell line LIM1863 by sequential centrifugal ultrafiltration are biochemically and functionally distinct. Methods..

[26] Greening DW, Xu R, Ji H, Tauro BJ, Simpson RJ (2015). A protocol for exosome isolation and characterization: evaluation of ultracentrifugation, density-gradient separation, and immunoaffinity capture methods. Methods Mol Biol..

[27] Sharma P, Ludwig S, Muller L, Hong CS, Kirkwood JM, Ferrone S, Whiteside TL (2018). Immunoaffinity-based isolation of melanoma cell-derived exosomes from plasma of patients with melanoma. J Extracell Vesicles..

[28] Jiang Z, Liu G, Li J (2020). Recent progress on the isolation and detection methods of exosomes. Chem Asian J..

[29] Tauro BJ, Greening DW, Mathias RA, Ji H, Mathivanan S, Scott AM, Simpson RJ (2012). Comparison of ultracentrifugation, density gradient separation, and immunoaffinity capture methods for isolating human colon cancer cell line LIM1863-derived exosomes. Methods..

[30] Muller L, Hong CS, Stolz DB, Watkins SC, Whiteside TL (2014). Isolation of biologically-active exosomes from human plasma. J Immunol Methods..

[31] Hiemstra TF, Charles PD, Gracia T, Hester SS, Gatto L, Al-Lamki R (2014). Human urinary exosomes as innate immune effectors. J Am Soc Nephrol..

[32] Lai RC, Arslan F, Lee MM, Sze NSK, Choo A, Chen TS (2010). Exosome secreted by MSC reduces myocardial ischemia/reperfusion injury. Stem Cell Res J..

[33] Liu F, Vermesh O, Mani V, Ge TJ, Madsen SJ, Sabour A (2017). The exosome total isolation chip. ACS Nano..

[34] Gámez-Valero A, Monguió-Tortajada M, Carreras-Planella L, Marcel-la F, Beyer K, Borràs FE. Size-exclusion chromatography-based isolation minimally alters extracellular vesicles’ characteristics compared to precipitating agents. Sci Reports. 2016;6:33641. 10.1038/srep33641 PMC5027519 27640641

[35] Sidhom K, Obi PO, Saleem A. A review of exosomal isolation methods: is size exclusion chromatography the best option? Int J Mol Sci. 2020;21(18):6466. 10.3390/ijms21186466 PMC7556044 32899828

[36] Vaidyanathan R, Naghibosadat M, Rauf S, Korbie D, Carrascosa LG, Shiddiky MJA, Trau M (2014). Detecting exosomes specifically: a multiplexed device based on alternating current electrohydrodynamic induced nanoshearing. Anal Chem..

[37] Zarovni N, Corrado A, Guazzi P, Zocco D, Lari E, Radano G (2015). Integrated isolation and quantitative analysis of exosome shuttled proteins and nucleic acids using immunocapture approaches. Methods..

[38] Bruce TF, Slonecki TJ, Wang L, Huang S, Powell RR, Marcus RK (2019). Exosome isolation and purification via hydrophobic interaction chromatography using a polyester, capillary-channeled polymer fiber phase. Electrophoresis..

[39] Wang L, Bruce TF, Huang S, Marcus RK (2019). Isolation and quantitation of exosomes isolated from human plasma via hydrophobic interaction chromatography using a polyester, capillary-channeled polymer fiber phase. Anal Chim Acta..

[40] Huang S, Wang L, Bruce TF, Marcus RK (2019). Isolation and quantification of human urinary exosomes by hydrophobic interaction chromatography on a polyester capillary-channeled polymer fiber stationary phase. Anal Bioanal Chem..

[41] Huang S, Ji X, Jackson KK, Lubman DM, Ard MB, Bruce TF, Marcus RK (2021). Rapid separation of blood plasma exosomes from low-density lipoproteins via a hydrophobic interaction chromatography method on a polyester capillary-channeled polymer fiber phase. Anal Chim Acta..

[42] Jackson KK, Powell RR, Marcus RK, Bruce TF (2022). Comparison of the capillary-channeled polymer (C-CP) fiber spin-down tip approach to traditional methods for the isolation of extracellular vesicles from human urine. Anal Bioanal Chem..

[43] Jackson KK, Marcus RK (2023). Rapid isolation and quantification of extracellular vesicles from suspension-adapted human embryonic kidney cells using capillary-channeled polymer fiber spin-down tips. Electrophoresis..

[44] Jackson KK, Mata C, Marcus RK (2023). A rapid capillary-channeled polymer (C-CP) fiber spin-down tip approach for the isolation of plant-derived extracellular vesicles (PDEVs) from 20 common fruit and vegetable sources. Talanta..

[45] Jackson KK, Powell RR, Bruce TF, Marcus RK (2021). Rapid isolation of extracellular vesicles from diverse biofluid matrices via capillary-channeled polymer fiber solid-phase extraction micropipette tips. Analyst..

[46] Wysor SK, Marcus RK (2023). Quantitative recoveries of exosomes and monoclonal antibodies from Chinese hamster ovary cell cultures by use of a single, integrated two-dimensional liquid chromatography method. Anal Chem..

[47] Billotto LS, Jackson KK, Marcus RK (2022). Determination of the loading capacity and recovery of extracellular vesicles derived from human embryonic kidney cells and urine matrices on capillary-channeled polymer (C-CP) fiber columns. Separations..

[48] Ji X, Huang S, Bruce TF, Tan Z, Wang D, Zhu Z (2021). A novel method of high-purity extracellular vesicle enrichment from microliter-scale human serum for proteomic analysis. Electrophoresis..

[49] Huang S, Wang L, Bruce TF, Marcus RK (2020). Evaluation of exosome loading characteristics in their purification via a glycerol-assisted hydrophobic interaction chromatography method on a polyester, capillary-channeled polymer fiber phase. Biotechnol Prog..

[50] Yu X, He L, Pentok M, Yang H, Yang Y, Li Z (2019). An aptamer-based new method for competitive fluorescence detection of exosomes. Nanoscale..

[51] Gercel-Taylor C, Atay S, Tullis RH, Kesimer M, Taylor DD (2012). Nanoparticle analysis of circulating cell-derived vesicles in ovarian cancer patients. Anal Biochem..

[52] Sokolova V, Ludwig A-K, Hornung S, Rotan O, Horn PA, Epple M, Giebel B (2011). Characterisation of exosomes derived from human cells by nanoparticle tracking analysis and scanning electron microscopy. Colloids Surf B..

[53] Dragovic RA, Gardiner C, Brooks AS, Tannetta DS, Ferguson DJ, Hole P (2011). Sizing and phenotyping of cellular vesicles using nanoparticle tracking analysis. Nanomed J..

[54] Soo CY, Song Y, Zheng Y, Campbell EC, Riches AC, Gunn-Moore F, Powis SJ (2012). Nanoparticle tracking analysis monitors microvesicle and exosome secretion from immune cells. Immunology..

[55] Kim YB, Lee GB, Moon MH (2022). Size separation of exosomes and microvesicles using flow field-flow fractionation/multiangle light scattering and lipidomic comparison. Anal Chem..

[56] Petersen KE, Manangon E, Hood JL, Wickline SA, Fernandez DP, Johnson WP, Gale BK (2014). A review of exosome separation techniques and characterization of B16–F10 mouse melanoma exosomes with AF4-UV-MALS-DLS-TEM. Anal Bioanal Chem..

[57] Kesimer M, Gupta R (2015). Physical characterization and profiling of airway epithelial derived exosomes using light scattering. Methods..

[58] Szatanek R, Baj-Krzyworzeka M, Zimoch J, Lekka M, Siedlar M, Baran J (2017). The methods of choice for extracellular vesicles (EVs) characterization. Int J Mol Sci..

[59] Randunu KM, Marcus RK (2012). Microbore polypropylene capillary channeled polymer (C-CP) fiber columns for rapid reversed-phase HPLC of proteins. Anal Bioanal Chem..

[60] Belliveau J, Papoutsakis ET (2022). Extracellular vesicles facilitate large-scale dynamic exchange of proteins and RNA among cultured Chinese hamster ovary and human cells. Biotechnol Bioeng..

[61] Skrika-Alexopoulos E, Smales CM (2023). Isolation and characterisation of exosomes from Chinese hamster ovary (CHO) cells. Biotechnol Lett..

[62] Cimorelli M, Nieuwland R, Varga Z, van der Pol E (2021). Standardized procedure to measure the size distribution of extracellular vesicles together with other particles in biofluids with microfluidic resistive pulse sensing. PLoS One..

[63] Anand S, Samuel M, Mathivanan S (2021). Exomeres: a new member of extracellular vesicles family. Subcell Biochem..

[64] Mathieu M, Martin-Jaular L, Lavieu G, Théry C (2019). Specificities of secretion and uptake of exosomes and other extracellular vesicles for cell-to-cell communication. Nat Cell Biol..

